# Death from Musical Excitement

**Published:** 1875-10

**Authors:** 


DEATH AS OCCASIONED BY MUSICAL EXCITEMENT.—A stout married countrywoman, some 28 years old, went to a festival where, for the first time in her life, says the *Repertorio del Piemonte*, she heard a powerful orchestra. The festival lasted three days, and she danced with much satisfaction during its whole continuance. When all was over she returned home, but the music, at least subjectively, returned with her. Do what she would, day and night and always, the music sounded in her ears. Nightly sweating and diarrhœa ensued. Medical interference proved fruitless, and at the end of six weeks, during all which time the imaginary orchestra never ceased for one minute, quite exhausted, the poor creature at length expired.—*The Doctor, Sept.* 1, 1875.

